# Identification of Monogenic Causes of Arterial Ischemic Stroke in Children with Arteriopathies by Next-Generation Sequencing

**DOI:** 10.3390/ijms26136228

**Published:** 2025-06-27

**Authors:** Anna Balcerzyk-Matić, Ilona Kopyta, Celina Kruszniewska-Rajs, Paweł Niemiec, Joanna Gola

**Affiliations:** 1 Department of Biochemistry and Medical Genetics, School of Health Sciences in Katowice, Medical University of Silesia in Katowice, Medykow 18, 40-752 Katowice, Poland; pniemiec@sum.edu.pl; 2 Department of Paediatric Neurology, Faculty of Medical Sciences in Katowice, Medical University of Silesia in Katowice, Medykow 16, 40-752 Katowice, Poland; ikopyta@sum.edu.pl; 3 Department of Molecular Biology, Faculty of Pharmaceutical Sciences in Sosnowiec, Medical University of Silesia in Katowice, Jednosci 8, 41-200 Katowice, Poland; ckruszniewska@sum.edu.pl (C.K.-R.); jgola@sum.edu.pl (J.G.)

**Keywords:** pediatric stroke, mutations, monogenic cause, next-generation sequencing

## Abstract

The leading causes of pediatric arterial ischemic stroke (PAIS) are arteriopathies, which refer to pathologies of the arterial walls in the brain. Since traditional risk factors for cardiovascular diseases in children play a smaller role than in adults, it can be supposed that genetic factors may be of particular importance in this age group. Therefore, this study aimed to identify mutations affecting the formation of vascular wall pathologies, which can subsequently lead to ischemic stroke. The study used a database of 92 Caucasian children diagnosed with ischemic stroke. From this group, 25 children with arteriopathies were selected. The study had an exploratory and descriptive design, with the aim of characterizing rare genetic variants in a selected cohort, without attempting formal statistical association testing. The sequencing was performed using the Illumina NextSeq 550 platform. A panel of 161 genes known to be associated with stroke or arteriopathies was selected for further analysis. We identified 10 pathogenic or likely pathogenic mutations in 15 patients. Among these, three are likely monogenic causes of stroke (*ELN*, *SCN5A*, and *VHL* genes), two are considered risk factors (*FV* and *ADAMTS13*), two have conflicting interpretations (*ACAD9* and *ENG*), and three are most likely benign (*CBS*, *PMM2*, and *PKD1*). The frequency of genetic variants underlying ischemic stroke or acting as risk factors for the disease in the studied group is significantly higher than the estimated frequency of monogenic forms of stroke in young adults and higher than in the general population. NGS testing is worth considering, especially in patients who exhibit certain symptoms that may suggest the presence of mutations.

## 1. Introduction

Acute cerebral ischemia in the pediatric population is a problem that occurs much less frequently than in adults [1]. However, due to the persistence of post-stroke deficits affecting more than half of the patients, as well as the high risk of recurrence and death in the acute phase of the disease, research on pediatric stroke is being conducted worldwide [2]. Cerebrovascular diseases remain among the ten most common causes of death in the pediatric population. The risk factors for stroke in children, including genetic conditions, differ from the known risk factors for ischemic stroke in the adult population.

In our previous studies on children after ischemic stroke, we searched for genetic polymorphisms that could be risk factors for the disease, including polymorphisms in genes related to the coagulation and fibrinolysis system, inflammation, and the metabolism of homocysteine or lipids [3,4,5]. The results of our research did not show that any of the studied polymorphisms significantly affected the risk of the disease in the studied population. This led us to consider whether the cause of the disease, at least in some cases, might be due to rare mutations rather than more common variants. It is estimated that in the population of young adults, about 5% of ischemic strokes are caused by monogenic diseases; however, due to their frequency, diagnostic difficulties, and significant phenotypic diversity, this number seems underestimated [6]. In children, this frequency is likely even higher because, in this age group, traditional risk factors for cardiovascular diseases, such as hypertension, lipid disorders, or smoking, play a smaller role than in adults. More than 50 monogenic diseases associated with stroke are known. Features that may suggest a genetic basis for stroke include young age at onset, family history, high recurrence rate, neurodevelopmental disorders present before stroke onset, other vascular disorders affecting the vascular bed of the eyes, skin, heart, or kidneys, as well as idiopathic headaches (mainly migraines), and the presence of vascular pathology detectable in neuroimaging studies [7,8,9].

Among the currently known and defined risk factors for stroke in children, the most common causes are arteriopathies, which are pathologies of the arterial walls in the brain. These can be congenital or acquired (most often post-inflammatory), and their course can be monophasic, stationary, transient, or progressive [10]. The incidence of arteriopathies in children with ischemic stroke is estimated by various authors to be 34–86%; the incidence has a strong connection to the region of the world where the research was conducted, as some arteriopathies have a genetic basis [11,12].

The research hypothesis proposed that ischemic stroke in children with the previously described additional features may be caused by monogenic diseases that lead to the development of arteriopathies and, consequently, the onset of acute brain ischemia. Therefore, the study aimed to determine the frequency and types of causative mutations in children after ischemic stroke with accompanying arteriopathies, as well as to analyze potential mutations in the context of factors that may suggest a genetic basis for the stroke (e.g., family history, neurodevelopmental disorders, recurrent stroke episodes). The study has an exploratory and descriptive design, with the aim of characterizing rare genetic variants in a selected cohort of pediatric patients with arteriopathies and stroke, without attempting formal statistical association testing.

## 2. Results

**Figure 1:**
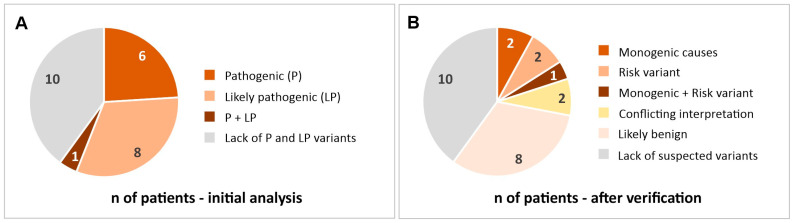
Number of patients with identified pathogenic and likely pathogenic variants before (**A**) and after verification (**B**).

The clinical characterization of a study group is shown in Table 1. Bioinformatic analysis identified 7786 genetic variants in the studied group, which were classified into the following categories: pathogenic, likely pathogenic, uncertain, likely benign, and benign (Table 2). We identified 10 different pathogenic or likely pathogenic mutations in 15 patients (some variants were present in more than one patient and some patients had more than one variant). Among these mutations, three are likely to be monogenic causes of stroke, two are considered risk factors for stroke, two have conflicting interpretations, and three are most likely benign (Figure 1). The results are summarized in Table 3 and Table 4.

### 2.1. Most Likely Monogenic Causes of Stroke

#### 2.1.1. ELN (Elastin)

The c.1577-2A>T mutation (rs1230920104) in the *ELN* gene was found in one of our patients in the heterozygous state. This mutation affects an acceptor splice site in intron 24 of the *ELN* gene and is expected to disrupt RNA splicing, which typically leads to a loss of protein function and is pathogenic. Elastin is an important component of the arterial wall, and one of the disorders caused by a heterozygous mutation in the *ELN* gene is supravalvular aortic stenosis, which manifests with arteriopathy, with multiple pulmonary and systemic arterial stenoses. The internal carotid artery (ICA) stenosis found in our patient appears to be consistent with the clinical symptoms of the disease. Another autosomal dominant disorder caused by mutations in the *ELN* is Cutis laxa typified by loose and/or wrinkled skin that imparts a prematurely aged appearance. In some cases, multiple vascular anomalies are present, including coarctation of the aorta and multiple peripheral pulmonary stenoses.

#### 2.1.2. SCN5A (Sodium Voltage-Gated Channel, Alpha Subunit 5)

One of our patients was heterozygous for the 655C>T, p.R219* (rs577421914) mutation in the *SCN5A* gene. This gene encodes the voltage-gated cardiac sodium channel SCN5A, which is responsible for the initial upstroke of the action potential in the electrocardiogram. Nonsense variant p.R219* leads to a premature termination codon, which is predicted to lead to a truncated or absent protein. Loss-of-function variants in the *SCN5A* gene are typically associated with a dominantly inherited Brugada syndrome-1 (characterized by ventricular arrhythmias and sudden cardiac death), although there can also be overlapping presentations, including Long QT syndrome [15]. However, the p.R219* variant is located in an exon that is part of a minor alternatively spliced transcript; therefore, its pathogenic role is suspected, but the clinical significance remains uncertain. This mutation is also likely pathogenic for Sick Sinus Syndrome 1 (sinus node dysfunction), but this is an autosomal recessive disease, while our patient is a heterozygote. Our patient had no abnormalities found in the ECG and echocardiogram through the chest. These tests, routinely performed in children after stroke, allow for the exclusion of most heart defects and cardiomyopathies. However, a single ECG does not allow for the exclusion of heart rhythm disorders or cardiomyopathies if they are in the early stages.

#### 2.1.3. VHL (Von Hippel–Lindau Tumor Suppressor)

One of our patients carried the c.439A>G, p.I147V mutation (rs1057517560) in the *VHL* gene in the heterozygous state. This is a tumor suppressor gene that plays a role in the oxygen-sensing pathway, microtubule stability and orientation, cilia formation, regulation of senescence, cytokine signaling, collagen IV regulation, and assembly of a normal extracellular fibronectin matrix [16]. Advanced modeling of protein sequence and biophysical properties performed at Invitae indicates that this missense variant is expected to disrupt VHL protein function with a positive predictive value of 80%; however, there are also conflicting predictions. Mutations in the *VHL* gene can cause Von Hippel–Lindau syndrome (VHLS), which is a dominantly inherited familial cancer syndrome predisposing to a variety of malignant and benign neoplasms, most frequently retinal, cerebellar, and spinal hemangioblastoma, renal cell carcinoma, pheochromocytoma, and pancreatic tumors. In the course of VHLS, pheochromocytomas tend to develop in the adrenal gland or paraganglia in children and young patients [16,17]. Some studies claim that paroxysmal hypertension and persistent arterial hypertension are the major symptoms of pheochromocytomas. Persistently high levels of arterial blood pressure may lead to the development of complications such as hypertensive cardiomyopathy, retinopathy, and retinal detachment. Other complications that may occur in the course of pheochromocytoma include myocardial infarction, heart failure, arrhythmia, stroke, and sudden cardiac death [18]. Our patient who carried this mutation was also a carrier of the *FV* c.1601G>A p. R534Q mutation (rs6025), which is described further and could be an additional factor having an impact on the occurrence of a stroke.

### 2.2. Mutations That Can Be a Risk Factor for Stroke

#### 2.2.1. FV (Coagulation Factor V)

Two of our patients carried the c.1601G>A, p.R534Q mutation of the *FV* gene (rs6025). It is referred to as factor V Leiden and was previously known as c.1691G>A, p.Arg506Gln. In vivo and in vitro functional studies provide evidence that the factor V Leiden variant impacts protein function and is a common cause of activated protein C resistance [19]. The factor V Leiden variant in a heterozygous state causes thrombophilia due to activated protein C resistance; however, homozygosity increases the risk of thrombotic complications to a greater extent. Heterozygosity for this variant is associated with a 6-to 8-fold increased risk for venous thrombosis [20]; however, clinical expression is variable, influenced by environment, and most individuals never develop thrombosis [21]. The heterozygous presence of the mutation may be combined with defects in other genes in the clotting pathway to contribute to the disorder, and this is likely the case in our patients.

#### 2.2.2. ADAMTS13 (A Disintegrin-like and Metalloprotease with Thrombospondin Type 1 Motif, 13)

ADAMTS13 is a protease that cleaves von Willebrand factor in circulating blood and thereby limits platelet thrombosis. Published functional studies demonstrate that the c.1370C>T, p.P457L variant (rs36220240) in the *ADAMTS13* gene found in one of our patients has a damaging effect, with impaired secretion and reduced enzyme activity [22]. In silico analysis supports that this missense variant has a deleterious effect on protein structure/function and is classified as likely pathogenic for Familial thrombotic thrombocytopenic purpura (hTTP), also known as Upshaw–Schulman syndrome. Acute phases of hTTP are defined by microangiopathic mechanical hemolytic anemia, severe thrombocytopenia, and visceral ischemia. Stroke occurs in 25% to 31% of patients [23]. This autosomal recessive thrombotic microangiopathy is caused mostly by biallelic mutation or, in very rare cases, by monoallelic *ADAMTS13* mutation associated with a cluster of single-nucleotide polymorphisms (USS; OMIM #274150). Although our patient was a heterozygote, he had five additional polymorphisms in this gene, so we cannot exclude that variation in this gene was the cause of abnormalities leading to a stroke. This mutation has also been reported as a single copy or an unspecified genotype in individuals with other unclear phenotypes such as congenital heart disease, acutely resolving episodes of hTTP, and in cohorts of individuals undergoing WES for rare bleeding disorders [22,24,25]. The genetic basis of our patient’s stroke may also be supported by the fact that his grandfather died of an ischemic stroke.

### 2.3. Variants with Conflicting Interpretations

#### 2.3.1. ACAD9 (Acyl-CoA Dehydrogenase Family, Member 9)

The c.976G>A, p.A326T mutation (rs115532916) of the *ACAD9* gene was found in a heterozygous state in two of our patients. Some homozygous or compound heterozygous mutations of this gene (e.g., c.976G>C) affect the structure and function of the ACAD9 protein and cause Mitochondrial complex I deficiency nuclear type 20 (MC1DN20). The symptoms are cardiorespiratory depression, hypertrophic cardiomyopathy, encephalopathy, and severe lactic acidosis. However, in the case of the c.976G>A mutation, there are conflicting classifications of pathogenicity, with most studies qualifying it as benign. On the other hand, one homozygous variant, *ACAD9* c.976G>A (p.Ala326Pro), was identified in the proband (stroke before 56 yo) of a family where two family members had developed stroke. Intracerebral bleeding was reported in two relatives, and one reportedly had hemispastic cerebral palsy of unknown cause. Additional phenotypes in this family included progressive muscle weakness starting in the early 40s, seizure episodes, scoliosis, and heart failure of unclear cause at adult age. Interestingly, the distribution of affected members in the pedigree was not easily compatible with recessive inheritance [26]. There is a possibility that there were compound heterozygous mutations. In our group, patient 137 has no other mutations in the *ACAD9* gene, and patient 52 has four additional polymorphisms qualified as benign. In summary, it seems that in our cases this mutation has no causal meaning, but it probably needs further investigation in the context of stroke.

#### 2.3.2. ENG (Endoglin)

The c.1633G>A, p.G545S mutation (rs142896669) in the *ENG* gene in a heterozygous state was found in one of our patients. This gene encodes a homodimeric transmembrane protein that is a major glycoprotein of the vascular endothelium. This protein is a component of the transforming growth factor beta receptor complex. Mutations in this gene cause Hereditary hemorrhagic telangiectasia (HHT), also known as Osler–Rendu–Weber syndrome 1, an autosomal dominant multisystemic vascular dysplasia. HHT leads to telangiectasia and arteriovenous malformations in the skin, mucosa, and viscera. Visceral involvement includes the lungs, liver, and brain. In association with HHT, mutation in the *ENG* gene can also cause Primary pulmonary hypertension 1 (PPH1)—a progressive vascular lung disease characterized by increased pulmonary vascular resistance and sustained elevation of mean pulmonary arterial pressure, leading to right ventricular hypertrophy and right heart failure. The c.1633G>A (p.(G545S)) mutation was initially identified in individual patients affected by pulmonary arterial hypertension [27] and pulmonary arterial hypertension associated with congenital heart defects [28]. Based on early analyses of this missense mutation using various software programs, the authors classified it as probably pathogenic [27]. However, further studies led to this variant being classified by ClinVar as likely benign due to its population frequency, presence in unaffected individuals, intact protein function, lack of segregation with disease, results of RNA analysis, in silico models, and amino acid conservation. Our patient’s mother died at the age of 30 due to renal artery aneurysms, and his grandfather died from an ischemic stroke, so a genetic basis is very likely; however, it seems that it should be sought among other genes.

### 2.4. Other Variants Found in a Study Group

#### 2.4.1. CBS (Cystathionine Beta-Synthase)

Mutations in the *CBS* gene, encoding cystathionine beta-synthase, can lead to enzyme activity deficiency and an autosomal recessive metabolic disorder known as classical homocystinuria. Some of its symptoms involve changes in the cardiovascular system, which can lead to stroke. Among our patients, four (16%) carried a c.832_833ins68 p.I278Tfs*16 mutation in the *CBS* gene, classified by the Franklin tool as likely pathogenic. Although it was present in a heterozygous form, there are many reported cases of classical homocystinuria occurring in compound heterozygotes [29], and each of our patients has at least one or more additional mutations/polymorphisms in the *CBS* gene. The variant found in our patients is most probably the equivalent of the previously described double mutation 833T>C, 844ins68, where the 68 bp insertion is present in cis with the 833T>C (I278T) mutation. In vitro studies and animal models show that the 833T>C (I278T) mutation leads to a reduction in enzyme activity [30]; thus, it is likely pathogenic. This heterozygous mutation has also been identified in a patient with early stroke and hyperhomocysteinemia, without other classical features of homocystinuria [31]. However, in the case of the double mutation 833T>C/844ins68, the insertion of 68 base pairs probably eliminates the 833T>C mutation through alternative splicing, thus abolishing its pathogenic effect [32]. This hypothesis seems to be supported by the relatively high frequency of this mutation, approximately 10%, in the Caucasian population [14]. In conclusion, it seems that the mutations detected in the *CBS* gene in our patients were not the cause of vascular abnormalities and stroke.

#### 2.4.2. PMM2 (Phosphomannomutase-2)

One of our patients was a c.422G>A (p.Arg141His) heterozygote in the *PMM2* gene (rs28936415). This missense variant is well-documented as the most common pathogenic variant found in patients with PMM2-associated congenital disorders of glycosylation (CDG-1a) [33]. Symptoms usually develop during infancy and may vary significantly among affected individuals. The most common manifestations include hypotonia, abnormal distribution of fat tissue, inverted nipples, developmental delay, cerebellar hypoplasia, and strabismus. Cases with thrombotic tendencies and cardiomyopathy have also been reported. This variant is typically observed in a compound heterozygous state in patients with severe phenotypes. However, our patient does not carry any other mutations in this gene, so in this case, it is probably benign. This patient had a congenital heart defect (common atrioventricular canal) and neurodevelopmental disorders, which may indicate a genetic basis; however, the cause should probably be sought in other genes.

#### 2.4.3. PKD1 (Polycystin 1)

Three of our patients had the c.971G>T p.R324L (rs199476099) mutation in the *PKD1* gene. Some mutations in this gene cause autosomal dominant Polycystic Kidney Disease type 1 (ADPKD), which can cause hypertension and significantly increase the risk of stroke [34]; however, there are conflicting interpretations of the pathogenicity of the c.971G>T mutation. Thomas et al. [35] identified this missense mutation in an adult patient with ADPKD, but the observed variant frequency within non-Finnish European control individuals in the gnomAD database is approximately eight times the estimated maximal expected allele frequency for a pathogenic variant in *PKD1* causing Polycystic Kidney Disease 1 phenotype. Additionally, three out of five in silico tools predicted a benign effect of the variant on protein function. Another argument against the causative role of this mutation in our group is that although the disease may manifest from infancy to 80 years, the typical age of onset is in middle adulthood. All these factors lead us to conclude that this variant is not a cause of stroke in the children we studied.

## 3. Discussion

In the studied group of 25 children with arteriopathies and ischemic stroke, mutations potentially underlying the disease were identified in three patients (12%).

One of them, the child with the ICA stenosis, had the c.1577-2A>T heterozygous mutation (rs1230920104) in the *ELN* gene. He probably suffered from autosomal dominant disorder supravalvular aortic stenosis, which manifests with arteriopathy, with multiple pulmonary and systemic arterial stenoses. Mutation in the *ELN* gene was previously associated with ischemic stroke in adult patients [36].

The second patient had a heterozygous mutation c.655C>T, p.R219* (rs577421914) in the *SCN5A* gene. Other mutations in this gene have been previously found in children with stroke [37]. Our 6-year-old patient underwent ECG and chest echocardiography, and there were no abnormalities found. The standard cardiac assessment for children with stroke involves conducting an ECG and chest echocardiography. This protocol allows the detection of pathologies such as congenital heart defects or cardiomyopathy. However, cardiac rhythm disorders may not be revealed during a single ECG examination. Knowing about specific point mutations predisposing to severe rhythm disorders, like long QT syndrome in a particular patient, would necessitate expanding diagnostic procedures, including Holter monitoring. Additionally, scheduling follow-up examinations every 6–12 months would be crucial if no abnormalities were detected initially. Baruteau et al. [38] also suggest that close follow-up and ICD implantation should be considered in symptomatic *SCN5A* mutation-positive children, even if displaying a negative ECG phenotype, because a substantial proportion of them will experience further recurrent events, even under appropriate treatment.

One of our patients carried the c.439A>G, p.I147V heterozygous mutation (rs1057517560) in the *VHL* gene, which causes dominantly inherited Von Hippel–Lindau Syndrome. VHLS patients are predisposed to develop lesions of the central nervous system and viscera, and the most common tumors in VHLS include hemangioblastomas [39]. In the course of VHLS, pheochromocytomas also tend to develop in the adrenal gland or paraganglia in children and young patients [17]. Some complications that may occur in the course of this disease include high arterial blood pressure, myocardial infarction, heart failure, arrhythmia, stroke, and sudden cardiac death. VHLS is considered a significant monogenic condition associated with pediatric stroke [40]. Our patient, who carried this mutation, had middle cerebral artery (MCA) stenosis and was also a carrier of the *FV* c.1601G>A, p.R534Q mutation (rs6025), which could be an additional factor contributing to the occurrence of a stroke.

None of the mentioned patients’ parents were found to have diseases caused by these genetic variants, suggesting that they were de novo mutations (e.g., about 20% of patients have VHLS as the result of a pathogenic variant that occurred as a de novo event in the affected individual or as a postzygotic de novo event in a mosaic, apparently unaffected parent [39]). It is important to note, however, that these were parents of young children and therefore relatively young and asymptomatic at the time of the stroke in their child, but this does not mean the absence of symptoms during the subsequent follow-up. There is also the possibility that in individuals with such mutations, the phenotypes can range from severe to mild and even asymptomatic, as was described by Li D [41] in cases involving *ELN* mutations and supravalvular aortic stenosis. In some families, phenotypes ranged from severe generalized obstructive disease requiring surgery at an early age to mild, asymptomatic, focal vascular abnormalities documented late in life through clinical testing.

Three of the analyzed patients (12%) had variants in genes that are not causative mutations but are known risk factors for ischemic stroke; these were mutations in the *FV* and *ADAMTS13* genes. Both genes are involved in the processes of coagulation and fibrinolysis, so they are likely not the cause of arteriopathy but rather may increase the risk of stroke by acting in a prothrombotic manner. Factor V Leiden increases the risk for venous thrombosis four- to eightfold when it occurs in a heterozygote state, while a mutation in *ADAMTS13* is likely pathogenic for familial TTP.

The next three patients had mutations in the *ACAD9* and *ENG* genes, the involvement of which in the disease cannot be excluded, but their significance remains unclear. Finally, in eight patients, mutations/polymorphisms were found, which, although classified by the tools we used as pathogenic or likely pathogenic, were considered probably insignificant after a further analysis. These were mutations in the *CBS*, *PMM2*, and *PKD1* genes. This confirms the need for a thorough analysis of each suspicious variant to avoid false positive results.

In the retrospective study by Kumar et al. [42] based on clinical exome sequencing, a high frequency of Mendelian disorders was also uncovered in children with stroke, particularly among infants. However, it is difficult to compare their findings directly with ours, as their cohort was less homogeneous and included patients with metabolic, prenatal, or hemorrhagic stroke or stroke associated with vessel dissection. Moreover, they conducted whole-exome analysis (except for a few cases where a panel of 5000 genes associated with Mendelian disease was used), whereas we analyzed a panel of selected genes. Interestingly, Kumar et al. [42] identified entirely different mutations than those found in our analysis, further highlighting the complex etiology of stroke in children.

According to the literature, features that may suggest a genetic basis for ischemic stroke in children and serve as indications for in-depth genetic diagnostics include a family history of the disease, neurodevelopmental disorders preceding the stroke, and recurrent strokes [6,7,8]. However, in our study group, these factors were not observed in any of the three patients with a causative mutation. This suggests that restricting the selection of patients for NGS testing to those exhibiting the above-mentioned factors might result in overlooking children who actually have a genetic basis for the disease.

On the other hand, in our study group we did not identify causative mutations in two patients with a clear positive family history of the disease, despite the unprecedented progress in knowledge about monogenic forms of stroke in recent years. In the case of adult stroke, the amount of information on this topic, published in PubMed and OMIM only between 2017 and 2022, has resulted in the expansion of the Stroke Gene Panel by more than 40% of genes [43]. Despite this, there is a suspicion that the panel of genes associated with adult stroke or arteriopathies may still be insufficient to identify the genetic causes of the disease in some children, which emphasizes the need to search for new genetic causative factors of childhood stroke. From a cognitive perspective, sequencing every pediatric stroke patient seems reasonable, as this approach could facilitate the development of a specialized gene panel for children with ischemic stroke while also determining the frequency and impact of specific mutations on the disease phenotype. Despite the financial investment required, NGS appears to be a more cost-effective approach to diagnosing ischemic stroke in children compared to single-gene analysis. By shortening the diagnostic process, it enables the identification of subpopulations of patients with similar mutations and the design and rapid implementation of personalized therapies targeted to specific groups of patients.

Due to the ultra-rare nature of the identified variants, conventional association testing with a control group was not performed. Instead, the frequency of monogenic causes of stroke in our cohort was compared with data available in the literature. To our knowledge, there are no existing data that define the frequency of monogenic diseases in children with idiopathic ischemic stroke. However, the frequency of genetic variants either causing ischemic stroke or acting as risk factors in our study group is significantly higher than the estimated frequency of monogenic forms of stroke reported in young adults [6]. The combined frequency of monogenic forms of stroke and risk variants in our group is also nearly nine times higher than in the general population (24% vs. 2.7%). Detailed knowledge about the frequency of monogenic causes of stroke in children would be very valuable for clinicians and could provide directions for selecting patients for in-depth genetic diagnosis using the NGS method.

In conclusion, the frequency of monogenic causes of ischemic stroke in children exceeds that observed in adults, and mutations also occur in patients who do not present many factors suggesting a genetic basis for the disease. Thus, further studies are needed to provide more detailed guidelines for clinicians regarding the use of NGS testing in the diagnosis of this condition. The increasing availability of NGS and decreasing analysis costs provide hope that, in the future, this method will be used to identify rare mutations responsible for ischemic stroke in children. This will enable more precise diagnoses and, consequently, more effective therapies, as suggested in the case of our patient with the *SCN5A* gene mutation.

### Limitations

Clinical data regarding patients undergoing genetic analysis are historical and were collected retrospectively. Neuroimaging studies were conducted in all children, but using various methods. For most children, follow-up studies were not performed, or if they were, they occurred shortly after the stroke event (within 14–30 days). This limitation means that although we have a diagnosis of arteriopathy, the course of the condition (stable, progressive, or reversible) cannot be determined. Therefore, according to current recommendations for diagnosis and follow-up of children with ischemic stroke, after the diagnosis of stroke and arteriopathy by one of the methods (MRI, MRA vs. CT, CTA), with MRI as the method of choice, it is necessary to carry out a follow-up examination 3–6 months after the stroke. Such a diagnostic scheme allows us not only to determine the cause of the stroke at the time of its occurrence (arteriopathy) but also its nature (stable, reversible, or progressive); this knowledge, in turn, would allow us to identify potential patients with a genetic background of arteriopathy, as reversible cases are often inflammatory and therefore acquired. Thus, when planning a prospective study, all children should have neuroimaging examinations performed at the same intervals and using the same method, optimally in one laboratory; standardizing the methodology and conducting it according to the same scheme allows for more precise conclusions. Also, data on diseases in the family—with particular attention to vascular diseases—requires a thorough questioning of the parents of a sick child about each member of the family.

## 4. Materials and Methods

### 4.1. Patient Cohort

The study used a database of 92 Caucasian children diagnosed with ischemic stroke based on a clinical examination and the results of additional tests, including neuroimaging. The cases were recruited among patients at the Upper Silesian Child Health Centre in Katowice and the Polish Mother’s Health Center Institute in Łódź, Poland, hospitalized between 2007 and 2009. Ethical approval for the study was obtained from the Bioethics Committee of the Medical University of Silesia in Katowice (NN-6501-64/07) and was performed in accordance with the ethical standards laid down in the 1964 Declaration of Helsinki and its later amendments. Written informed consents were submitted by the parents of the patients. The current study used only previously collected clinical data from hospital patient records and biological material (blood) stored at the Department of Biochemistry and Medical Genetics of the Medical University of Silesia in Katowice. Due to the retrospective nature of the clinical data collection and the time elapsed since patient recruitment, the scope of neuroimaging studies performed at that time, both in the acute phase and during follow-up, differed from the currently known models and recommendations [44,45,46].

**Figure 2:**
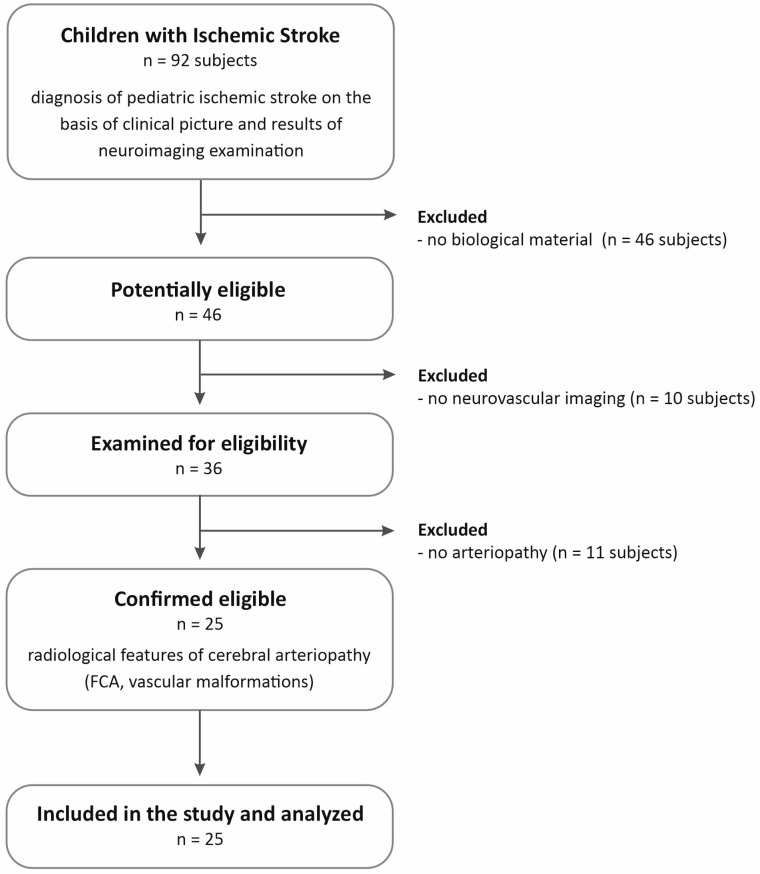
The flowchart presents the criteria of patient recruitment for the study. Legend: FCA—focal cerebral arteriopathy of childhood.

From the group of 92 patients, 25 children were selected who, according to medical data, underwent neuroimaging examinations during the acute phase of the disease, and the results indicated arteriopathy. Children in whom stroke was confirmed by any neuroimaging test (magnetic resonance imaging, MRI) were included in the study, while vascular imaging was conducted using magnetic resonance angiography (MRA) or Transcranial Doppler (TCD) flow studies. The patient selection scheme is presented in Figure 2.

The inclusion criteria were as follows:

1. Age from 29 days of age to the completion of 18 years of age
2. Diagnosis of pediatric arterial ischemic stroke (PAIS) based on the clinical picture and results of neuroimaging examination. PAIS was defined as an acute neurological deficit with sudden onset occurring in a child between the 29th day of life and 18 years of age, and the vascular cause of the symptoms observed in the patient is confirmed by neuroimaging studies, and the location of the changes in the neuroimaging studies corresponds to the clinical symptoms.
3. Radiological features of cerebral arteriopathy (focal cerebral arteriopathy of childhood (FCA) or other vascular malformations)
4. Caucasian origin

In addition, from the data in the patients’ files, we collected information on other diseases with a potential vascular origin in the patient (migraine, idiopathic headache), vascular pathologies in organs other than the CNS (skin, heart and large vessels, kidneys, other internal organs), cardiovascular diseases in the family (parents, siblings, grandparents), recurrent stroke in the patient, and neurodevelopmental disorders occurring in the child before the stroke.

Exclusion criteria were as follows:

5. Age below 29 days or above 18 years
6. Lack of neuroimaging studies
7. No confirmed diagnosis of ischemic stroke
8. Diagnosis of a CNS pathology other than ischemic stroke (stroke mimics)
9. Lack of biological material

### 4.2. Next-Generation Sequencing (NGS)

The peripheral blood samples were used for DNA isolation, which was performed using the MasterPure™ genomic DNA purification kit (Epicentre Technologies) according to the manufacturer’s instructions. Quantification of genomic DNA was performed using a Quantus fluorimeter with the QuantiFluor^®^ ONE dsDNA System (Promega, Madison, WA, USA). Library preparation and sequencing were performed according to the manufacturer’s instructions (Illumina, San Diego, CA, USA). A total of 250 ng of DNA was used to prepare dual-indexed paired-end libraries using the Illumina DNA Prep with Enrichment workflow (Illumina, San Diego, CA, USA). The TruSight One Panel Kit (Illumina, San Diego, CA, USA) was used for sequencing the coding regions of over 4800 genes associated with clinically relevant phenotypes.

The protocol included the following steps: tagmentation of genomic DNA, post-tagmentation clean-up, amplification of tagmented DNA, pre-enriched libraries clean-up, probe hybridization and capture of hybridized probes, amplification, and clean-up of the enriched library. Quality assessment of pre-enriched and enriched libraries was performed using the Agilent Technology 2100 Bioanalyzer (Agilent, Santa Clara, CA, USA).

Sequencing of the generated library was performed using the Illumina NextSeq 550 platform in accordance with the manufacturer’s instructions. Data obtained from the sequencing were uploaded to BaseSpace (Illumina, San Diego, CA, USA) for the generation of the VCF files using the DRAGEN Enrichment App. Sequencing reads were aligned to the GRCh38 (Genome Reference Consortium Human Build 38) human reference genome. Mean region coverage depth and coverage uniformity (Pct > 0.2 × mean) were 158× and 95.2%, respectively.

### 4.3. Definition of Gene Panel and Genetic Analysis

A panel of 161 genes confirmed to be associated with the occurrence of stroke or arteriopathies, was selected for further analysis. The list of genes was compiled based on data from the HPO—The Human Phenotype Ontology database (phenotype: Stroke—HP:00012970) and literature data on monogenic forms of arteriopathy and ischemic stroke in children [47,48,49,50]. Variant calling and annotation were performed using bioinformatic applications—Franklin (Genoox, Tel Aviv, Israel) [13] and BaseSpace Variant Interpreter (Illumina, San Diego, CA, USA) [51]. The Franklin platform uses an artificial intelligence-driven engine designed to prioritize and interpret variant data and to classify mutations as pathogenic, likely pathogenic, of uncertain significance, likely benign, or benign. This engine compiles evidence from various sources, including public databases, literature-based resources, exclusive Franklin Community data, and in-house curation. Franklin’s AI-based classification engine adheres to the ACMG/AMP standards and guidelines [52]. The BaseSpace Variant Interpreter was used to verify and supplement the data from Franklin. In the search for clinically significant genetic variants, we focused exclusively on pathogenic and likely pathogenic variants. The evaluation of these variants included assessing their frequency, predicted impact on protein function, and potential clinical significance (based on data from ClinVar—a public archive of reports on the relationships among human variations and phenotypes, gnomAD—the Genome Aggregation Database, and OMIM—Online Mendelian Inheritance in Man) [53,54]. Due to the ultra-rare nature of the identified variants, adjustment for potential confounding factors was not performed, since conventional association testing does not apply in this case.

## Figures and Tables

**Table 1:** Characteristics of the group selected for the NGS study.

	Mean (Min.–Max.)
Age at stroke onset (in months)	83.9 (1–214)
	n (%)
Sex	male 13 (52) female 12 (48)
Imaging methods used in the acute phase of stroke:	
only MRI, MRA	15 (60)
angiography + MRI, MRA	3 (12)
only TCD	3 (12)
TCD + MRI	4 (16)
Other vascular diseases	1 (4)—TIA 2 weeks before stroke
Diseases located in a vasculature beyond the CNS	1 (4)—congenital heart defect (common atrioventricular canal)
Neurodevelopmental disorders before stroke	4 (16)
Recurrence of stroke	1 (4)
Cardiovascular diseases in the child’s family	2 (8)

Legend: MRI—magnetic resonance imaging, MRA—magnetic resonance angiography, TCD—Transcranial Doppler, CNS—central nervous system, TIA—transient ischemic attack.

**Table 2:** Variant type proportions within the studied cohort (based on the Franklin by Genoox [13]).

The Number of Each Variant Type				
Pathogenic	Likely pathogenic	Uncertain	Likely benign	Benign
7 *	9 *	175	145	7450

* The numbers of pathogenic and likely pathogenic variants differ from the numbers mentioned in the text because some variants were present in more than one patient and some patients had more than one variant.

**Table 3:** Monogenic causes of stroke and mutations that are the risk factors for stroke.

No.	Gene	Genotype	Mutation		MAF (gnomAD)	Phenotype Generally Associated (Inheritance)	Phenotypic Features
			c.DNA	AAC			
1	*ELN* (LP)	Het	c.1577-2A>T	-	0.000	Supravalvular Aortic Stenosis (AD) Autosomal Dominant Cutis Laxa (AD)	- arteriopathy, with multiple pulmonary and systemic arterial stenoses - loose and/or wrinkled skin, in some cases multiple vascular anomalies including coarctation of the aorta, multiple peripheral pulmonary stenoses, including stenoses in very small vessels
2	*SCN5A* (P)	Het	c.655C>T	p.R219*	0.000	Brugada syndrome (AD) Sick Sinus Syndrome 1 (AR)	- ventricular arrhythmias and sudden cardiac death - sinus node dysfunction, syncope, presyncope, dizziness, and fatigue
3	*VHL* (LP) *FV* (P)	Het Het	c.439A>G c.1601G>A	p.I147V p.R534Q	0.000 0.025	Von Hippel–Lindau syndrome (VHLS) (AD) Factor V deficiency (AR) Thrombophilia due to activated protein C resistance (AD)	- familial cancer syndrome predisposing to a variety of malignant and benign neoplasms, e.g., pheochromocytoma developing in the adrenal gland or paraganglia and causing chronic hypertension, which can result in cardiomyopathy, myocardial infarction and heart failure, arrhythmia, stroke, and sudden cardiac death - prolonged bleeding, hemorrhagic diathesis tendency to thrombosis
4	*FV* (P)	Het	c.1601G>A	p.R534Q	0.025	Factor V deficiency (AR) Thrombophilia due to activated protein C resistance (AD)	- prolonged bleeding, hemorrhagic diathesis tendency to thrombosis
5	*ADAMTS13* (P) *ACAD9* (P)	Het Het	c.1370C>T c.976G>A	p.P457L p.A326T	0.002 0.02	Familial thrombotic thrombocytopenic purpura (AR) Mitochondrial complex I deficiency, nuclear type 20 (AR)	- microangiopathic mechanical hemolytic anemia, severe thrombocytopenia, visceral ischemia - cardiorespiratory depression, hypertrophic cardiomyopathy, encephalopathy, severe lactic acidosis

Legend: P—pathogenic, LP—likely pathogenic, AAC—amino acid change, AD—autosomal dominant, AR—autosomal recessive, MAF—minor allele frequency (from Ensembl for the European population, the frequency of CBS c.832_833ins68 mutation is from the literature [14]).

**Table 4:** Variants with conflicting interpretations and likely benign.

No.	Gene	Genotype	Mutation		MAF (gnomAD)	Phenotype Generally Associated (Inheritance)	Phenotypic Features
			c.DNA	AAC			
1	*ENG* (P)	Het	c.1633G>A	p.G545S	0.000	Hereditary hemorrhagic telangiectasia (AD) Primary Pulmonary Hypertension in association with HHT	- telangiectases and arteriovenous malformations of skin, mucosa, and viscera (lung, liver, brain) - increased pulmonary vascular resistance and sustained elevation of mean pulmonary arterial pressure, right ventricular hypertrophy and right heart failure
2	*PMM2* (P)	Het	c.422G>A	p.R141H	0.006	Congenital disorders of glycosylation (AR)	- severe encephalopathy with axial hypotonia, abnormal eye movement, pronounced psychomotor retardation, peripheral neuropathy, cerebellar hypoplasia, retinitis pigmentosa, peculiar distribution of subcutaneous fat, nipple retraction, hypogonadism, cardiomyopathy
3	*PKD1* (LP)	Het	c.971G>T	p.R324L	0.004	Polycystic Kidney Disease type 1 (AD)	- renal cysts, liver cysts, intracranial aneurysm, and hypertension
4	*CBS* (LP)	Het	c.832_833ins68	p.I278Tfs*16	0.1 ^a^	Classic homocystinuria (AR)	- myopia, ectopia lentis, intellectual disability, skeletal anomalies, and thromboembolic events
5	*ACAD9* (P)	Het	c.976G>A	p.A326T	0.02	Mitochondrial complex I deficiency, nuclear type 20 (AR)	- cardiorespiratory depression, hypertrophic cardiomyopathy, encephalopathy, and severe lactic acidosis

Legend: P—pathogenic, LP—likely pathogenic, AAC—amino acid change, AD—autosomal dominant, AR—autosomal recessive, MAF—minor allele frequency (from Ensembl for the European population), ^a^ the frequency of CBS c.832_833ins68 mutation is from the literature [14].

## Data Availability

Sequencing data generated in this study have been deposited in the NCBI SRA database with the primary accession code PRJNA1159500.

## Abbreviations

The following abbreviations are used in this manuscript:

AAC	amino acid change
ACAD9	Acyl-CoA dehydrogenase family, member 9
AD	autosomal dominant
ADAMTS13	A disintegrin-like and metalloprotease with thrombospondin type 1 motif, 13
ADPKD	autosomal dominant Polycystic kidney disease type 1
AR	autosomal recessive
CBS	cystathionine beta-synthase
CDG-1a	congenital disorders of glycosylation
CNS	central nervous system
ELN	Elastin
ENG	Endoglin
FCA	focal cerebral arteriopathy of childhood
FV	coagulation factor V
HHT	Hereditary hemorrhagic telangiectasia
hTTP	Familial thrombotic thrombocytopenic purpura
LP	likely pathogenic
MAF	minor allele frequency
MC1DN20	Mitochondrial complex I deficiency nuclear type 20
MRA	magnetic resonance angiography
MRI	magnetic resonance imaging
NGS	next-generation sequencing
P	pathogenic
PKD1	Polycystin 1
PMM2	Phosphomannomutase-2
PPH1	Primary pulmonary hypertension 1
SCN5A	sodium voltage-gated channel, alpha subunit 5
TCD	Transcranial Doppler
TIA	transient ischemic attack
VHL	Von Hippel–Lindau tumor suppressor
VHLS	Von Hippel–Lindau syndrome
